# A Suppression Method of Concentration Background Noise by Transductive Transfer Learning for a Metal Oxide Semiconductor-Based Electronic Nose

**DOI:** 10.3390/s20071913

**Published:** 2020-03-30

**Authors:** Huixiang Liu, Qing Li, Zhiyong Li, Yu Gu

**Affiliations:** 1 School of Automation and Electrical Engineering, University of Science and Technology Beijing, Beijing 100083, China; liuhuixiang@xs.ustb.edu.cn (H.L.); liqing@ies.ustb.edu.cn (Q.L.); 2 College of Computer Science and Electronic Engineering, Hunan University, Changsha 410082, China; zhiyong.li@hnu.edu.cn; 3 Key Laboratory for Embedded and Network Computing of Hunan Province, Changsha 410082, China; 4 Beijing Advanced Innovation Center for Soft Matter Science and Engineering, Beijing University of Chemical Technology, Beijing 100029, China; 5 Department of Chemistry, Institute of Inorganic and Analytical Chemistry, Goethe-University, Max-von-Laue-Str. 9, 60438 Frankfurt, Germany

**Keywords:** drift suppression, dimensionality reduction, electronic nose, domain adaptation, transfer learning

## Abstract

Signal drift caused by sensors or environmental changes, which can be regarded as data distribution changes over time, is related to transductive transfer learning, and the data in the target domain is not labeled. We propose a method that learns a subspace with maximum independence of the concentration features (MICF) according to the Hilbert-Schmidt Independence Criterion (HSIC), which reduces the inter-concentration discrepancy of distributions. Then, we use Iterative Fisher Linear Discriminant (IFLD) to extract the signal features by reducing the divergence within classes and increasing the divergence among classes, which helps to prevent inconsistent ratios of different types of samples among the domains. The effectiveness of MICF and IFLD was verified by three sets of experiments using sensors in real world conditions, along with experiments conducted in the authors’ laboratory. The proposed method achieved an accuracy of 76.17%, which was better than any of the existing methods that publish their data on a publicly available dataset (the Gas Sensor Drift Dataset). It was found that the MICF-IFLD was simple and effective, reduced interferences, and deftly managed tasks of transfer classification.

## 1. Introduction

In recent years, as a reliable, time-saving, and cost-efficient technique, the Electronic Nose (E-nose) has been applied in many fields, including aided medical diagnosis [1,2], food engineering [3], environmental control [4,5], and explosive detection [6]. Specifically, Metal Oxide Semiconductor (MOS) gas sensors, which have the advantage of cross-sensitivity, broad spectrum response, and low-cost, have been widely used in conjunction with the E-nose [7]. Identification of an unknown odor is the core content of the research and application of the E-nose. However, in the general environment, adaptive identification is very difficult due to the inherent inconsistency (e.g. sensitivity, selectivity, and reversibility) of the manufacturing process of sensors. As a dynamic process, signal drift, caused by poisoning, aging, or environmental changes, is one of the most important defects of gas sensors [8], and it reduces the selectivity and sensitivity of gas sensors. Once the sensor’s signal drifts, the input–output relations built at the calibration phase will be destroyed, rendering the classification or regression model invalid for the E-nose and diminishing its practicality. It has not been possible to create a gas sensor without drift because of the limitations of the relevant technology. 

Strategies that are frequently used to solve the problem of sensor drift within the chemical sensing field may be either univariate or multivariate, and include methods in which drift compensation is performed either on each sensor individually or on the entire sensor array, such as baseline calibration [9] and component correction [10,11]. There are several drawbacks to these methods. For example, they require extra reference gas to approximate the drift direction by assuming that the drift tendency of each sensor is the same. Ensemble learning, an important branch of machine learning, has attracted much attention and has been used to cope with sensor drift [12,13]; it can improve the generalization performance of the learning algorithm by building and combining multiple learners. Although this type of approach automatically adapts classifier to drift, it cannot calculate or explicitly describe the drift [14]. In addition, one must use the label of the target data (to be tested) during the process of model training, which is insignificant in practice. With the improvement and development of machine learning theories, transfer learning has recently become a focus in the field of computer vision [15], and it is also used to address the problem of sensor drift [16,17,18]. The ideal solution based on transfer learning will avoid expensive data-labeling efforts and thus greatly enhance learning performance, given that no labeled data in the target domain is available while much labeled data in the source domain is available [19].

In this paper, we extend the understandings of Hilbert–Schmidt independence criterion (HSIC) and feature extraction method based on Fisher Linear Discriminant (FLD), to improve the transferring capability and generalization of a general-purpose machine learning method among multiple domains, with very few labeled guide instances in the target domain. Motivated by the idea of “domain features” [18], we first defined “concentration features” for the samples to rank the concentration information. Then, a latent feature space was found, in which the samples are mostly independent of the concentrations, in terms of the HSIC [20]. However, for data which is collected from long-term measurement processing, it was difficult to eliminate all the interference of the drift only by means of the HSIC-based model. We think that the differences which are caused by the sensor’s self-drift (without concentration interference) still exist among domains. The drift caused by unequal concentration is the main factor for the gas classification, but the sensor’s self-drift (self-aging, long-term drift, etc.) could not be overlooked. Then, we minimized the within-class scatter while maximizing the between-class scatter, from source domain to target domain based on the FLD, to further reduce the differences of data distribution among domains. 

The remainder of this paper is organized as follows. Section 2 introduces related work on HSIC-based feature extraction and unsupervised domain adaptation. Concentration features and the Fisher development criterion are described in Section 3 in detail. In Section 4, we show the experimental process in detail and analyze the experimental results. Conclusions are drawn in Section 5.

## 2. Related Work

### 2.1. Maximum Independence of Domain Feature

Researchers have aimed to reduce the dependence between the extracted features and the domain feature (or device feature) using the Hilbert–Schmidt Independence Criterion (HSIC) [18]. According to reference [20], the estimation of the HSIC is shown as follows. Let Z = {(x_1_, y_1_), ……, (x_n_, y_n_)} ⊆X×Y be a series of n independent samples drawn from p_xy_. An estimation of HSIC, written as HSIC (Z, F, G), is defined as:

(1) $$\mathrm{HSIC}(Z,F,G)={\left(m-1\right)}^{-2}\mathrm{tr}(KHLH),$$

where *H*, *K*, *L*∈*ℝ*^n×n^, *K*_ij_ = k(x_i_, x_j_), and *L*_ij_ = k(y_i_, y_j_) represent the kernel matrices associated with RKHSs F and G, respectively, and *H* = **1** − n^−1^**11**^T^. For suitable kernels, HSIC (p_xy_, F, G) = 0 if, and only if, **x** and **y** are independent [21].

### 2.2. Unsupervised Domain Adaptation

The transfer learning methods generally can be divided into three categories: (1) instance-based methods, (2) model-based methods, and (3) feature-based methods [22]. We have given more attention to feature-based methods, which attempt to preserve important properties (geometric structure and statistical properties), or to reduce the discrepancy of distribution across domains. Maximum Mean Discrepancy (MMD) was used to evaluate any dissimilarity across the empirical distributions of the source and target domains [23]. For example, Transfer Component Analysis (TCA) searches a latent space in which the variance of the data is preserved as much as possible, and the distance in the marginal distribution is reduced across domains [24]. Long et al. proposed a joint distribution adaptation method (JDA) to match both marginal and conditional distribution between domains [25]. However, these methods ignore the differences in class proportion between different domains. Geodesic flow kernel (GFK) is another popular method used for domain adaptation; it represents the feature space from the viewpoint of differential geometry, considering the subspaces along a path of geodesic flow from the source domain to the target domain [16]. The main disadvantage of GFK is that the constructed subspaces do not represent the original data accurately, because they select a small dimension for smooth transit across flow [22].

### 2.3. Fisher Linear Discriminant (FLD)

FLD, as a supervised learning method, has been widely applied in the fields of statistics, pattern recognition, and machine learning. The goal of FLD is to find a linear transformation, such that the within-class scatter matrix, S_w_, is minimized, and the between-class scatter matrix, S_b_, is maximized. The transform matrix, *V*, can be computed as:

(2) $$\underset{V\in \;R^{n\times k}}{\arg\;\max}\;J=\frac{V^{T}S_{b}V}{V^{T}S_{w}V},$$

where $S_{w}=\sum_{Z_{i}}^{Z_{N}}\sum_{z\in Z_{i}}(z-\mu_{i}){\left(z-\mu_{i}\right)}^{T}$ refers to the within-class scatter matrix; $S_{b}=\sum_{i=1}^{N}\lambda_{i}(\mu_{i}-\mu ){\left(\mu_{i}-\mu \right)}^{T}$ is the between-class scatter matrix, in which *μ_i_* is the mean vector in *Z_i_*, and *μ* is the mean vector of all of the samples; *λ_i_* is the weight of samples marked class *i*; and *N* is the total number of categories.

In general, Equation (2) can be equivalent to:

(3) $$\underset{V^{T}S_{w}V=I}{\max}tr(V^{T}S_{b}V)$$

and according to the Lagrange multiplier theory, Equation (3) could be simplified as:

(4) $$S_{b}V=\lambda S_{w}V.$$

Finally, the problem of finding the optimal transform matrix, *V*, is simplified to the solution of Equation (4) for the k largest eigenvectors.

## 3. Proposed Method

As we know, when drift exists, the gas measurement or identification result may cause errors. Sensor drift is inevitable when a device runs continuously for long periods. In this study, we think that the drift caused by unequal concentration is a main interference factor for the gas classification. However, the sensor’s self-drift (self-aging, long-term drift, etc.) could not be overlooked, especially in long-term measurement processing. Users have difficulty determining whether the signal drift is caused by inconsistent concentration or by the drift of the sensor itself, especially if the concentration of the sample to be tested is unknown. Therefore, the factors that cause sensor signal drift consists of two parts: the concentration factor and the sensor’s self-drift. In order to suppress signal drift, we proposed the MICF-IFLD method. Firstly, the concentration interference is suppressed based on the Maximum Independence of the Concentration Features (MICF), and we then eliminate the sensor’s self-drift using the Iterative Fisher Linear Discriminant (IFLD).

### 3.1. Maximum Independence of the Concentration Features (MICF)

We proposed the MICF method according to the HSIC. Here, we considered that the concentration of the gasses used for training impacts the performance of the classifier in the class task of target domain (a detailed analysis will be shown in Section 4). Thus, we seek to extract a novel feature that is as independent as possible from the concentration information. First, a set of “concentration features” was designed to describe and rank the concentration information. We denote *X*∈ℝ^m×n^ as the matrix of n samples, which consisted of training samples and testing samples, and had a concentration range from tens to thousands of ppm. According to the subject of this study, no tag information (class and concentration) was available in the testing samples. 

We define $d_{j}=\ln c_{j}$, j=1, …, n, where c_j_ is the concentration of **x**_j_, and the integer portion of d_j_ is considered the concentration level. The concentration features of matrix Y are set up as follows. For c-levels of concentration, the analogous one-hot coding scheme can be borrowed; i.e., *Y*∈ℝ^c×n^, y_ij_ = I if **x**_j_ is labeled as concentration level I, and I =1, ……, c; y_ij_ = 0. Otherwise, **x**_j_ belongs to the testing samples. The linear kernel function is selected for the concentration kernel matrix as follows:

(5) $$K_{y}=Y^{T}Y.$$

For mapping X to a new space, a linear or nonlinear mapping function, Φ, is needed. The exact form of Φ is not required based on the kernel trick, and the inner product of Φ(X) can be acquired by the kernel matrix K_x_ = Φ(X)^T^Φ(X). Like other kernel dimensionality reduction algorithms [18,26], the matrix *W*∈ℝ^n×h^ (h ≤ m) makes the following equation true:

(6) $$Z=W^{T}K_{x}.$$

Intuitively, if the projected features are independent of the concentration features, then we believe that the concentration is no longer affecting the fingerprint of the projected features, which indicates that the concentration discrepancy in the subspace decreases. As a result, after omitting the scaling factor in Equation (1), our goal is to find an orthogonal transformation matrix *W*∈ℝ^n×h^ such that HSIC (Z, X, Y) is minimized:

(7) $$\underset{W^{T}W=I}{\min}\mathrm{tr}(K_{z}HK_{y}H)=\underset{W^{T}W=I}{\min}\mathrm{tr}(K_{x}WW^{T}K_{x}HK_{y}H)$$

where K_z_ represents the kernel matrix of Z.

In transductive transfer learning, the goal is to minimize the difference in data distributions and to preserve important properties of the original data. According to variance maximization theory, this can be achieved by maximizing the trace of the covariance matrix of the project samples. The covariance matrix is:

(8) $$\mathrm{cov}\;(Z)=\mathrm{cov}\;(W^{T}K_{x})=W^{T}K_{x}HK_{x}W,$$

where $H=I-n^{-1}1_{n}1_{n}^{T}$. An orthogonal constraint is further added to W. The learning problem then becomes:

(9) $$\underset{W^{T}W=I}{\max}\mathrm{tr}(-W^{T}K_{x}HK_{y}HK_{x}W+\mu W^{T}K_{x}HK_{x}W),$$

where µ > 0 is a trade-off hyper-parameter. Using the Lagrangian multiplier method, we can find that W represents the eigenvectors of $K_{x}\left(-HK_{y}H+\mu H\right)K_{x}$ corresponding to the *h* largest eigenvalues. 

For computing the kernel matrices, K_x_ and K_y_, some common kernel functions are available, including the Linear function, the Polynomial function, and the Gaussian Radial Basis function.

### 3.2. Iterative Fisher Linear Discriminant (IFLD)

It is particularly noteworthy that Z is a mixed samples matrix consisting of samples from both source domain and target domain where class labels are unavailable. To apply FLD across domains, we first train a benchmark classifier only on the source domain, and we can then predict the labels of the testing samples of the target domain. Thus, if we use these labels as the pseudo target labels and run FLD iteratively, which we define as IFLD, we will gradually improve the authenticity of these pseudo target labels until convergence. The effectiveness of the pseudo label refinement procedure, which is similar to the Expectation-Maximization (EM) algorithm, is verified by experiments. The complete algorithm of the proposed MICF-IFLD is presented as follows.
**Algorithm:** MICF-IFLD**Input**: The matrix of all samples *X*; class labels and concentration information of the training samples. **Output:** The adaptive matrix, *V*, in Equation (4), the classifier, *f*. **Begin**  Step 1: Construct the concentration features according to MICF.  Step 2: Compute the kernel matrices, *Kx* and *Ky*.  Step 3: Obtain orthogonal transformation matrix, *W*; namely, the eigenvectors of    $K_{x}\left(-HK_{y}H+\mu H\right)K_{x}$ corresponding to the *h* largest eigenvalues; *Z*
*= W^T^.;*  Step 4: Train the classifier based on the labeled features of the projected samples, Z. **Repeat**  Step 5: Construct with-class scatter matrix, *S_w_*, and between-class scatter matrix, *S_b_.*  Step 6: Compute transformation matrix, *V*, according to Equation (4). **Until** Convergence.  Step 7: Return to the adaptive matrix, *V*, and classifier, *f*.
**End**

## 4. Experiments

### 4.1. Experiments in the Authors Laboratory

**Figure 1:**
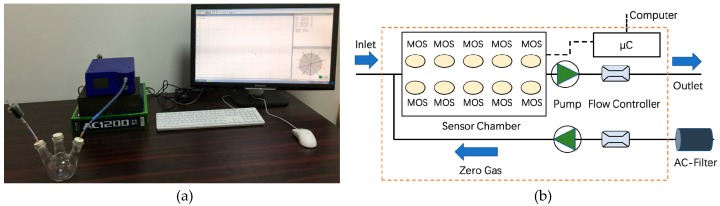
Experimental setup: (**a**) experimental equipment and (**b**) internal structure of PEN3 [27].

We used a dataset that was recorded by a PEN3 E-nose (Airsense Analytics GmbH, Germany [27], Figure 1) to verify the proposed method. The key part of the E-nose is a sensor array consisting of 10 different metal-oxide-semiconductor (MOS) sensors. Table 1 lists the details of the 10 sensors used in the experiments [27].

First, we prepared ethanol solutions and n-propanol solutions at concentrations of 10%, 20%, 33%, 50%, 67%, 80%, and 90%. Then, for each kind and concentration, three samples were measured every three days, and the experiments lasted for 45 days. All experiments were conducted in the authors laboratory (temperature: 25 ± 1℃, relative humidity: 50 ± 2%).

**Figure 2:**
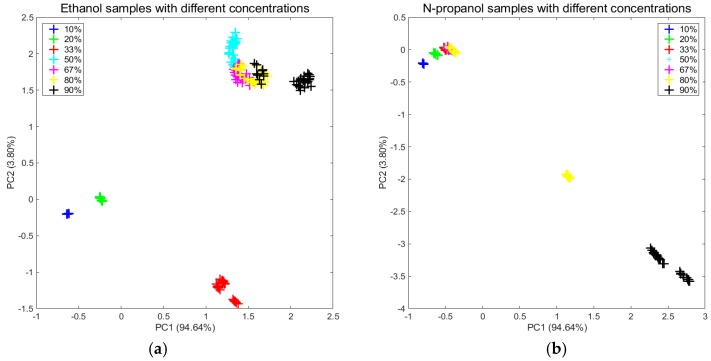
Projections of the two first primary components of the PCA computed for the two original datasets: (**a**) ethanol and (**b**) n-propanol.

**Figure 3:**
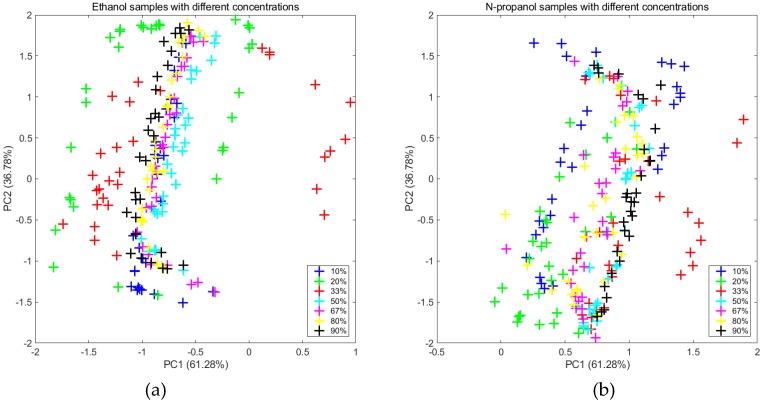
Projections of the two first primary components of the PCA computed for the two new feature datasets which were transformed from the original data by the MICF.: (**a**) ethanol and (**b**) n-propanol.

From the measurements on the first day, as shown in Figure 2a,b, the two subgraphs clearly display that the distributions significantly changed in terms of concentration. By extracting new features (a set of data which was transformed from the original data by the MICF) that were independent of the concentrations, it was obvious that the distributions tended to be uniform, which are shown in Figure 3a,b. 

In addition, we compare the classification performance of the original features (raw data) and MICF by means of a BP network classifier for the two kinds of samples. The training set was built from the measurements tested on the first day, and the testing set was based on the last day. It was found that the accuracy of classification increased from 73.81% to 87.62% after using MICF, as compared to the original features. Therefore, the various distributions of the measurements caused by different concentrations is a major obstacle in terms of the qualitative identification of gasses conducted by the MOS-based E-nose. From the above experiments, it was found that concentration is an important factor that cannot be neglected in the area of gas classification.

### 4.2. Experiments on a Publicly Available Gas Sensor Drift Dataset 

#### 4.2.1. Gas Sensor Drift Dataset and Experiments Set

The gas sensor drift dataset is a popular publicly available dataset created by Vergara et al. [12,28], which is used in pattern recognition for gas analysis. The dataset was employed in our experiments to evaluate the performance of the proposed algorithm for dealing with gas classification tasks that involve sensor drift. The dataset is created over a period of three years, and it gathers 13,910 records measured from 16 metal-oxide gas sensors that are positioned in six gasses with different concentrations. The possible gas type-concentration pairs are all sampled in a random order. Each sample data includes class label, concentration, and a 128-dimension feature vector (16 sensors, and each sensor contains two steady-state features and six transient features).

Table 2 details the dataset. Samples are split into 10 batches according to their acquisition time to ensure a sufficient number of samples in each batch. The goal of our experiment was to identify the types of gasses as accurately as possible, ignoring the interference of data drift.

As we learned from [12], the samples in batch 1 were used as labeled training samples, and those in batches 2–10 were considered unlabeled testing samples. In other words, the measurements of the labeled samples in batch 1, which we considered to be closest to the true values (data without drift), was divided into source domain samples. The unlabeled samples in the other batches (batch 2, 3, …, 10) were adopted as the target domains. To evaluate the generalization performance of the proposed method, the model was trained only in the source domain and tested in several target domains. Before the experiment, we normalized each feature to a zero mean first, and recorded the unit variance of each batch.

In this three-year Gas Sensor Drift Dataset, although the drift caused by unequal concentration was the main interference factor for the gas classification, the sensor’s self-drift (self-aging, long-term drift, etc.) could not be overlooked. In order to strengthen the generalization ability of the MICF-based model (solving the sensor’s self-drift problem), we proposed an IFLD method, and combined it with the MICF (named MICF-IFLD) to further improve the accuracy of gas classification.

#### 4.2.2. The Effect of Concentration on Data Distributions

For a given MOS sensors array, fingerprints formed by the same gas with different concentrations were not consistent because of the non-linear relationship and broad-spectrum response of the sensors, resulting in a difference in fingerprints, which can be regarded as the data distribution variations in the feature space.

**Figure 4:**
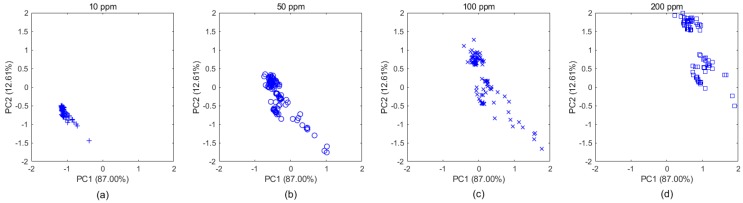
Example of the data drift caused by concentration in Batch 10. The Figure 4a–d show that the original data of ethylene samples (with 4 different concentrations) in Batch 10 is projected into two dimensional subspaces, whose basis is two principal components computed by PCA. The drift causes the distribution of ethylene data in the same batch to be different from the different concentrations.

**Figure 5:**
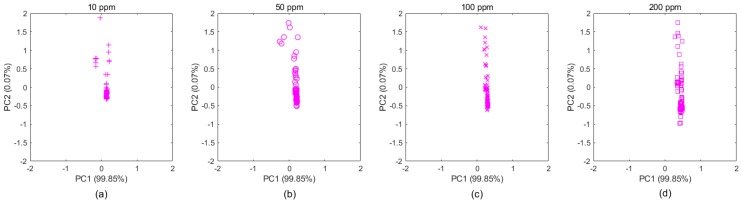
Example of the new data transformed by the Maximum Independence of the Concentration Features (MICF) in Batch 10. The Figure 5a–d show that the new ethylene data, transformed from the original data by the MICF, is projected into two dimensional subspaces, whose basis is two principal components computed by PCA. The drift interference caused by concentration was suppressed, so the data distributions tended to be consistent.

Taking the ethylene samples in Batch 10 as an example, as shown in Figure 4, the subgraphs a–d clearly display the distribution changes over concentration. These samples belong to the same category, even though their distributions appear to be different due to different concentrations, which makes gas classification more difficult. Therefore, for gas classification, concentration is an important factor that should be considered, regardless of which domain the samples came from. By extracting new features that were independent of concentrations based on the MICF, we found that the distributions tended to be consistent, which is shown in Figure 5a–d.

**Figure 6:**
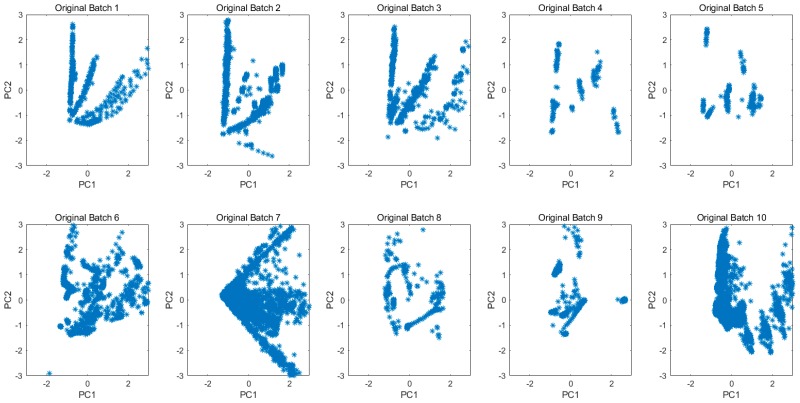
Principal component space of 10 batches for all original data.

**Figure 7:**
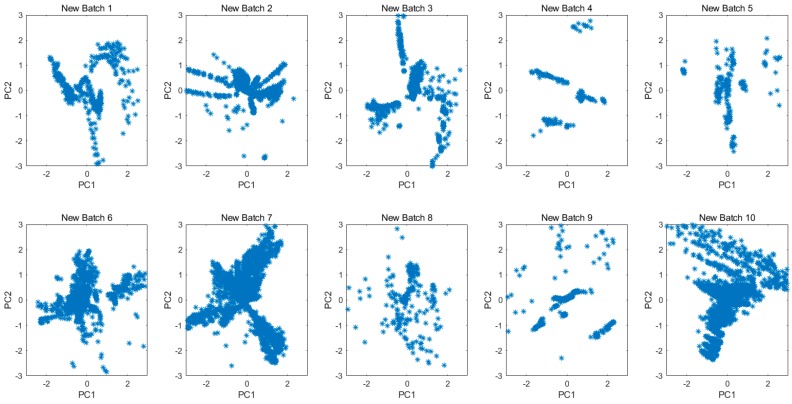
Principal component space of 10 batches for all new data which were transformed from original measurements by the MICF.

Not limited to the gas concentrations or the samples kinds, the projected 2D subspace for all original data in each batch is shown in Figure 6. The drift is clearly demonstrated by the different data distributions among batches. Figure 7 shows the principal component space of 10 batches for all new data which were transformed from original measurements by the MICF. The results of the data distribution were not as good as we expected, because the MICF-based model is poor at solving the problem of long-term gas measurement. It is necessary to consider the interference of the long-term drift of sensors. In order to solve the long-term drift of sensor, we proposed the IFLD-based model, and combined it with the MICF-based model to further improve the accuracy in the gas classification task. The long-term drift will be overcome based on the IFLD during the classification model training.

#### 4.2.3. Suppression of Sensors Drift Based on MICF-IFLD

In terms of comparisons, several mainstream machine learning methods were employed to classify the samples with original features, including Random Forest (RF) [29], eXtreme Gradient Boosting (XGBoost) [30], Support Vector Machine (SVM) [31], and Back Propagation Neural Network (BPNN). Other transfer learning (domain adaptive)-based methods, such as TCA [19], Comgfk-ml [14], and SMIDA [18], were also referenced. Based on new projected sample features obtained by the MICF and the MICF-IFLD, we used the BPNN as the classifier, and the classification accuracy was used to evaluate the methods’ performance.

Table 3 shows the experimental results. Seven methods were compared, including the mainstream machine learning methods and transfer learning-based methods. In general, the MICF and the MICF-IFLD obtained significant improvements in accuracy for several of the batches, and the MICF-IFLD had the highest accuracy among all the methods. From Table 3, we can draw the following conclusions:

Compared with mainstream machine learning methods without transfer learning, overall, the MICF and the MICF-IFLD performed significantly better than the other methods. The single MICF model can effectively reduce the interference caused by the concentration in the gas classification task, and the fusion MICF-IFLD model presents the best results over several iterations between the source domain and the target domain.

Additionally, the performance of all participating methods in Table 3 degrades over time because of the distance between the target domain and the source domain, which leads to the gradual increase of drift measures. Therefore, the MICF-IFLD can improve the performance and reduce the recalibration rate, but the method still cannot fundamentally resolve the sensor drift.

### 4.3. Experiments on the Application of Commercial Chinese Liquor Classification

#### 4.3.1. Experimental Samples and Experiment Setup

The measurements were carried out on three different kinds of commercial Chinese liquor samples (their details were shown in Table 4) provided by Hunan Xiangjiao Liquor Industry Co., Ltd., over one year to verify the impact of sensor drift on the online measurements. For each kind of liquor, samples came from three production lots. For each kind and each lot, 30 samples were considered for experimentation. Five samples were tested every two months; that is, 45 samples from all samples were tested every two months. The experiments lasted for one year (12 months) and 270 samples in total were measured. In other words, the same kind and lot of samples had been stored for different numbers of days when they were tested. As we know, Chinese liquor is produced by the blending of basic liquor, and the concentrations of the liquor vary from different production lots [32]. More notably, even for the same kind and lot of liquor, the alcohol content is slightly different due to technical or measurement error, and the alcohol content slowly decreases as the storage time increases. In order to fully verify the MICF-IFLD method we proposed, our goal is to distinguish between the three different kinds of the liquors, regardless of their lots, and to overcome data drift. Only the data tested in the first two months was used to train the classification model, and the subsequent measured data was used for testing separately. 

We used the same PEN3 E-nose that was mentioned in Section 4.1 for our experiments. The size of the raw dataset for the E-nose was 3 (kinds) × 3 (lots) × 5 (samples) × 6 (two months) which equaled 270 samples. The measured data for each of the two months was named chronologically as Batch 1, …, Batch 6, respectively. All experiments were conducted in the authors’ laboratory (temperature: 25 ± 1℃, relative humidity: 50 ± 2%).

#### 4.3.2. Experimental Results Based on MICF and MICF-IFLD

**Figure 8:**
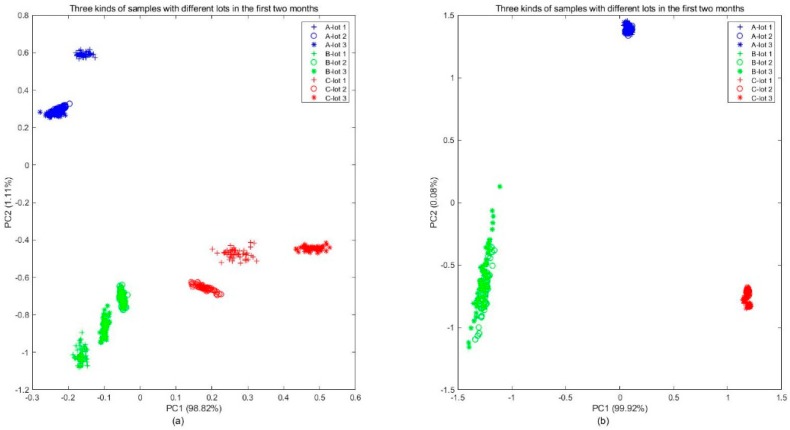
Examples of distribution varying across different kinds and lots: (**a**) original measurements and (**b**) new features that are independent of lots extracted from original measurements by the MICF.

The 45 samples in Batch 1 were analyzed by PCA, as shown in Figure 8a, and the scatters clearly displayed the distribution difference over kinds and lots. From the plot, it was found that while different kinds of samples were clustered in different regions, the distribution of samples with different lots in the same category were still slightly different. For manufacturers and consumers, the difference between different lots should be ignored. By extracting new features that are independent of lots using the MICF, it was obvious that the distributions of the different kinds of samples tended to be separable, and the distributions of the different lots tended to be uniform, which was shown in Figure 8b.

**Figure 9:**
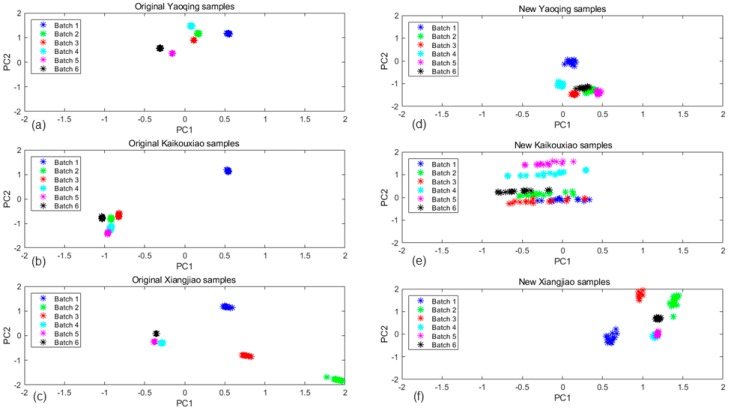
Projections of the two first primary components of the PCA computed for original data and new data which were transformed by the MICF: (**a**) original Yaoqing samples data, (**b**) original Kaikouxiao samples data, (**c**) original Xiangjiao samples data, (**d**) new Yaoqing samples data, (**e**) new Kaikouxiao samples data, and (**f**) new Xiangjiao samples data.

By showing each batch data changing over time, we compared all batches of sample data (six batches in a year) distributions before and after implementing by the MICF, which were shown in Figure 9. The three subgraphs (9a,b,c) represent the original data distributions of the three types of liquors, respectively. The Figure 9d,e,f represent the data distributions after the MICF transforming. It was found that the proposed MICF model can also effectively suppress cross-domain drift.

We tried to identify the three kinds of liquors as accurately as possible, while suppressing the data drift caused by the different production lots and the passage of time. The drift suppression experiments were performed in the gas classification task based on the MICF-IFLD method that we proposed. Additionally, the samples in Batch 1 were labeled and used for training, while those in Batches 2–6 were testing samples without labeling.

Table 5 shows the experimental results, in which the number indicates the number of correctly classified samples in the 45 testing samples of each batch. We found that the MICF-IFLD model achieves the highest average accuracy among all the methods. Compared with several other popular methods (BPNN, SVM, RF) without transfer learning, the MICF-IFLD performed significantly better overall. Although the performance of the four methods listed in Table 5 degrades over time, the proposed method can improve performance and decrease the recalibration rate, which provides theoretical guidance for solving the drift problem of similar instruments in long-term online applications.

**Figure 10:**
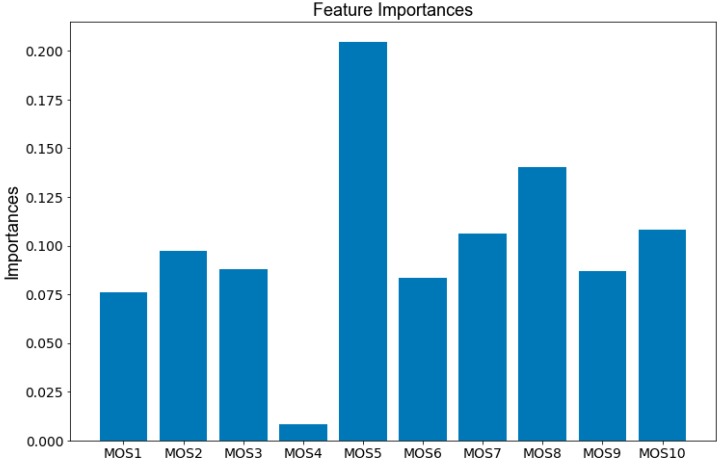
Importance ranking of each sensor in the E-nose.

The contribution rate of each sensor in the E-nose in the classification task is shown in Figure 10, from which we can see that the fifth MOS sensor has the largest contribution for distinguishing those three types of liquors.

## 5. Conclusions

In this paper, we demonstrate the negative effect of concentration features in gas classification tasks. The method maps a subspace that has the maximum independence of the concentration features based on the HSIC criterion, which reduces the inter-concentration discrepancy among the distributions. Our experiments show that MICF effectively improves the performance of the gas classification task. 

To reduce the drift caused by the sensor itself or by the environment, the IFLD was used to further extract the signal features by reducing divergence within each class and increasing divergence among classes, which helps to prevent inconsistent ratios of different types of samples among the domains. Combined with the IFLD, the accuracy of the classification was further improved. It was found that the MICF-IFLD method was simple and effective, and that this method achieved the best accuracy (76.17%) as compared with the existing methods based on the Gas Sensor Drift Dataset. Because of the simplicity and effectiveness of the MICF-IFLD, this method could reduce interference caused by the sample itself while dealing with the tasks of transfer classification.

The MICF-IFLD suppression method of concentration background noise is proposed in this study. It improves the robustness of the prediction model for interference suppression when using the MOS-based E-Nose.

## Figures and Tables

**Table 1:** The details of sensor array in PEN3 [27].

Number	Sensor	Object Substances for Sensing
MOS1	W1C	aroma constituent
MOS2	W5S	sensitive to nitride oxides
MOS3	W3C	ammonia, aroma constituent
MOS4	W6S	hydrogen
MOS5	W5C	alkane, aroma constituent
MOS6	W1S	sensitive to methane
MOS7	W1W	sensitive to sulfide
MOS8	W2S	sensitive to alcohol
MOS9	W2W	aroma constituent, organic sulfur compounds
MOS10	W3S	sensitive to alkane

**Table 2:** Dataset details.

Batch No.	Months	Etha	Ethy	Ammo	Acetal	Acet	Tolu	Total
1	1–2	90	98	83	30	70	74	445
2	3–4, 8–10	164	334	100	109	532	5	1244
3	11–13	365	490	216	240	275	0	1586
4	14–15	64	43	12	30	21	0	161
5	16	28	40	20	46	63	0	197
6	17–20	514	574	110	29	606	467	2300
7	21	649	662	360	744	630	568	3613
8	22–23	30	30	40	33	143	18	294
9	24, 30	61	55	100	75	78	101	470
10	36	600	600	600	600	600	600	3600

**Table 3:** Accuracy of classification obtained by the experiment results (%).

	Batch 2	3	4	5	6	7	8	9	10	Average
RF	82.07	76.92	62.11	74.62	52.26	43.54	53.4	32.55	28.61	56.23
XGBoost	84.81	80.01	63.98	78.68	63.48	53.42	56.46	37.45	32.44	61.19
SVM	87.78	77.68	57.14	73.60	62.35	47.91	46.94	32.77	32.25	57.60
BPNN	88.26	78.18	59.63	73.10	55.70	43.15	55.78	35.32	34.92	58.23
TCA	82.96	81.97	65.22	76.14	89.09	68.98	49.32	66.17	49.50	68.82
Comgfk-ml	80.25	74.99	78.79	67.41	77.82	71.68	49.96	50.79	53.79	67.28
SMIDA	83.68	82.28	73.91	75.63	93.00	63.49	79.25	62.34	45.50	72.23
MICF	88.91	83.04	68.94	91.88	83.35	58.04	60.88	56.81	47.86	71.08
MICF-IFLD	95.18	84.05	71.87	93.99	89.54	63.93	65.48	66.50	54.97	76.17

**Table 4:** The details of Chinese liquors in the experiment.

No.	Chinese Liquors	Flavors	Proof	Date of Production	Place of Origin	Price/Bottle ($)
A	Yao Qing	Nong Jiang	101.6	Lot 1,2017.9, Lot 2,2018.1, Lot 3,2018,3,	Shaoyang, Hunan, China	205
B	Kai Kouxiao-Jiu Nian	Strong	101.6	Lot 1,2017.9, Lot 2,2018.1, Lot 3,2018,3,	Shaoyang, Hunan, China	28
C	Xiang Jiao-Hong Zuan	Nong Jiang	101.6	Lot 1,2017.9, Lot 2,2018.1, Lot 3,2018,3,	Shaoyang, Hunan, China	115

**Table 5:** Accuracy of classification obtained by BPNN, SVM, RF, and MICF-IFLD (number of correctly classified samples and accuracy).

	Batch 2	Batch 3	Batch 4	Batch 5	Batch 6	Average
BPNN	40(88.89%)	36(80.00%)	30(66.67%)	27(60.00%)	25(55.56%)	31.6(70.22%)
SVM	39(86.67%)	34(75.56%)	32(71.11%)	26(57.78%)	25(55.56%)	31.2(69.33%)
RF	37(82.22%)	34(75.56%)	33(73.33%)	29(64.44%)	23(51.11%)	31.2(69.33%)
MICF-IFLD	41(91.11%)	38(84.44%)	34(75.56%)	33(66.67%)	29(64.44%)	35(77.78%)
